# Augmented Renal Clearance in a Case of Sepsis Leading to Vancomycin Failure Despite Increasing Dose As per the Estimated Glomerular Filtration Rate

**DOI:** 10.7759/cureus.14183

**Published:** 2021-03-30

**Authors:** Rama Kanth Pata, Chhabilal Bastola, Nway Nway, Meet J Patel, Samaj Adhikari

**Affiliations:** 1 Pulmonary Medicine, Interfaith Medical Center, Brooklyn, USA; 2 Internal Medicine, Interfaith Medical Center, Brooklyn, USA

**Keywords:** vancomycin, augmented renal clearance, arc, sepsis, mrsa

## Abstract

Augmented renal clearance (ARC) is a unique clinical scenario observed in critically ill patients. We present a case of a 30-year-old male with sepsis secondary to methicillin-resistant Staphylococcus aureus (MRSA) bacteremia treated with vancomycin. ARC was observed in the patient with a maximum estimated glomerular filtration rate (eGFR) of 161.9 ml/min/1.73 m^2^, and therapeutic drug monitoring was used to adjust the vancomycin dosage. Despite the maximal recommended dose of vancomycin, the therapeutic vancomycin level was not achieved, leading to treatment failure and subsequent mortality. Our case report suggests the necessity of other strategies, such as early dose adjustment of vancomycin based on vancomycin clearance and continuous vancomycin infusion, not merely conventional adjustment based on eGFR and vancomycin levels.

## Introduction

Dosing of drugs in intensive care has a huge impact on outcomes. Frequently, dosing is adjusted based on pharmacokinetics, which is dependent on many factors and not just limited to co-morbidities and rapidly changing hemodynamics. Active vigilance should be exercised in liaison with a dedicated pharmacist; more importantly, for those drugs with a narrow therapeutic window and those medications whose efficacy depends on the therapeutic level. In an intensive care setting, anticoagulation and antibiotics require therapeutic drug monitoring. Vancomycin-induced renal toxicity requiring hemofiltration has been reported in the intensive care setting [1-2]. Most commonly, antibiotics are adjusted based on the degree of reduction of eGFR from baseline to prevent toxicity. On the contrary, an increase in eGFR would result in therapeutic failure. Augmented renal clearance (ARC) is a unique phenomenon encountered in critically ill patients, resulting in the enhanced elimination of solutes, higher than expected for age, clinical co-morbidity, or gender. The phenomenon of ARC was documented in the 1970s in burn patients who required higher doses of aminoglycosides and was attributed to renal hyperfiltration [3]. ARC is considered when creatinine clearance exceeds 130 ml/min/1.73m^2^ [4-5]. ARC has been reported among severely septic patients, traumatic brain injury, and young and healthy individuals who have undergone surgery or multiple trauma [6-7]. Many predictive scoring systems and tools are used to predict ARC. A failure to do early projection can lead to antibiotic failure resulting in mortality. We present a case of a critically ill patient with ARC secondary to methicillin-resistant Staphylococcus aureus (MRSA) sepsis, resulting in vancomycin failure and mortality.

## Case presentation

A 30-year-old male (weight 72.5 kg and height: 6 ft 3-inch, body mass index (BMI) of 20.0 kg/m^2^) with a past medical history of intravenous drug use and schizophrenia presented to the emergency department with progressive shortness of breath, fever, and chills for three days. At presentation, his temperature was 102 °F, heart rate of ~120/min, respiratory rate ~30-35 breaths/min, and oxygen saturation of 93%-94% on room air. Physical examination revealed bilateral crepitation on the bases of the lungs. Complete blood count was significant for neutrophilia, and a preliminary diagnosis of sepsis was made. Table 1 shows the laboratory workup on admission.

**Table 1:** Laboratory workup on admission Hco3^-^: serum bicarbonate; BUN: blood urea nitrogen; AST: aspartate transaminase; ALT: alanine transaminase; ALP: alkaline phosphatase; mg/dl: milligram per deciliter; mmol/l: millimoles per liter; U/L: unit per liter

Tests	Results	Normal values	Tests	Results	Normal values
Lactic acid	2.8 mmol/l	0.5-1.9 mmol/l	Calcium	8.6 mg/dl	8.4-10.2 mg/dl
Sodium	134 mmol/l	136-145 mmol/l	Phosphorus	4.8 mg/dl	2.3-4.7 mg/dl
Potassium	3.4 mmol/l	3.5-5.1 mmol/l	Magnesium	1.7 mg/dl	1.6-2.6 mg/dl
HCO3-	26 mEq/L	22-29 mEq/L	Total bilirubin	1.8	0.2-1.2 mg/dl
Anion gap	10	8-16	AST	55 U/L	5-34 U/L
BUN	12.9 mg/dl	8.4-25.7 mg/dl	ALT	64 U/L	10-55 U/L
Creatinine	0.96 mg/dl	0.72- 1.25 mg/dl	ALP	42 U/L	40-150 U/L
Glucose	152 mg/dl	70-105 mg/dl	Total protein	5.6 gm/dl	6.0-8.3 gm/dl

**Figure 1:**
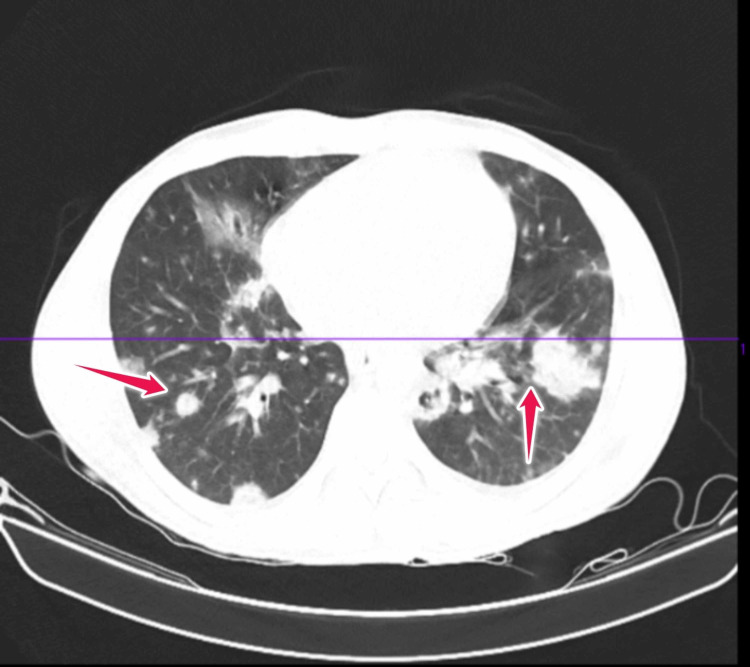
CT scan of chest done at the time of admission CT: computed tomography

CT chest (Figure 1) revealed innumerable different-sized irregular-shaped patchy and nodular densities in bilateral lungs (red arrows), some of them with central cavitation. Based on clinical manifestations and radiologic imaging, sepsis secondary to pneumonia was diagnosed.

In view of intravenous drug use, there was a high index of suspicion of MRSA sepsis. The patient was empirically treated with meropenem 1 gm every eight hourly and vancomycin 1 gm every 12 hourly to have synergistic action against MRSA sepsis.

Bronchoscopy was done, and bronchoalveolar lavage (BAL) revealed MRSA. Meropenem was discontinued on Day 4 when MRSA was isolated. Later on, vancomycin was continued for MRSA sepsis. The patient required endotracheal intubation owing to hypoxemic respiratory failure.

Vancomycin was started on Day 1 with 1 gram every 12 hours (27.7 mg/kg). After three initial doses, the vancomycin trough was 5.8 mg/dl. The critical care pharmacy was consulted, and vancomycin dosing was changed to 1 gm every eight hours (42.8 mg/kg). On Day 4, the vancomycin level was 9.6 mg/dl, so the dose of vancomycin was increased further to 1 gram every six hours (55.5 mg/kg). On Days 5 and 6, he received a total of 4.25 gm per day (~59mg/kg) of vancomycin. Despite these doses, the patient was not able to reach the therapeutic vancomycin levels. Consultation with the infectious disease team was done and the antibiotic was switched to ceftaroline and daptomycin. Linezolid was not considered in view of possible bone marrow suppression [8].

**Figure 2:**
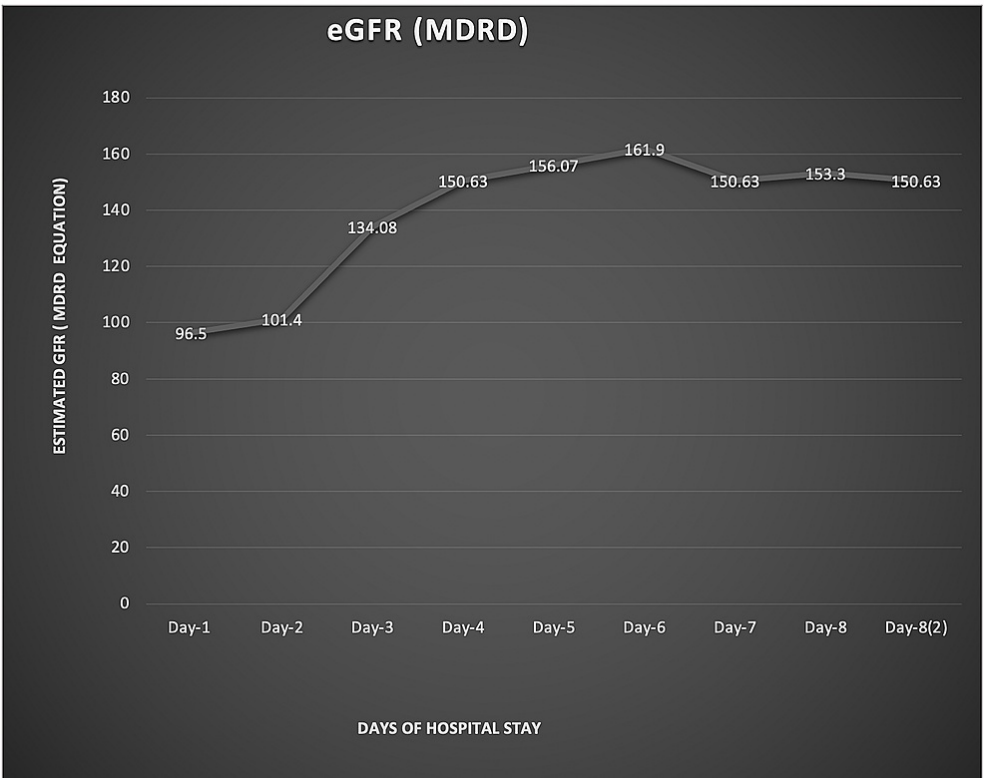
Estimated glomerular filtration rate (eGFR) trend based on the Modification of Diet in Renal Disease (MDRD) study equation MDRD: Modification of Diet in Renal Disease; eGFR: estimated glomerular filtration rate

Table 2 shows the vancomycin dose adjustment based on eGFR while Figure 2 shows the estimated glomerular filtration rate (eGFR) trend based on the Modification of Diet in Renal Disease (MDRD) study equation.

**Table 2:** Vancomycin dose adjustment based on eGFR eGFR: estimated glomerular filtration rate; ml/min: milliliter per minute; m^2^: meter square; mg/kg: milligram per kilogram; gm: gram; q12 HR: every 12 hourly; q8 HR: every eight hourly; q6 HR: every six hourly; HR: hour

eGFR	Vancomycin dose
96.5 ml/min/1.73 m2	1 gm Q12 HR (27.7mg/kg)
101.4 ml/min/1.73 m2	1 gm Q8 HR (42.8 mg/kg).
134.08 ml/min/1.73 m2	1 gm Q8HR (42.8 mg/kg).
150.63 ml/min/1.73 m2	1 gm Q6HR (55.5 mg/kg).
161.9 ml/min/1.73 m2	4.25 gm day in 4 divided doses (59 mg/kg)

ARC was recorded on Day 3 (134.08 ml/min/1.73 m2) and reached the highest value of 161.9 ml/min/1.73 m^2^ on Day 6. Despite the maximum recommended dose of vancomycin, there was a failure to achieve the therapeutic vancomycin level of ~ 15-20 mg/dl. Unfortunately, despite all efforts, the patient had worsening partial pressure of carbon dioxide/fraction of inspired oxygen (PaCO_2_/FiO_2_) ratio with severe acute respiratory distress syndrome (ARDS), and the patient expired on the eighth day of hospital stay.

## Discussion

The incidence of ARC ranges from 4.1% to 32.8% in studies done in intensive care settings [4,9]. Neuro-hormonal, temperature, vascular changes like cerebral autoregulation, fluid resuscitations, alteration of the physiology of nephrons, including but not limited to higher glomerular filtration, and variable tubular secretion and reabsorption have been suggested as the underlying pathophysiology of ARC [10-11]. Vancomycin being hydrophilic and excreted by up to 90% in urine in unchanged form, it is evident that vancomycin clearance depends on renal functioning and the phenomenon of ARC has a significant impact on [12].

Table 3 depicts recommended vancomycin interval dosing adjustment based on the eGFR level [7].

**Table 3:** Recommended vancomycin as per ARC stratification Mahmoud SH, Shen C: Augmented Renal Clearance in Critical Illness: An Important Consideration in Drug Dosing. Pharmaceutics. 2017, 9:10.3390/pharmaceutics9030036 [7] ARC: augmented renal clearance; eGFR: estimated glomerular filtration rate

ARC stratification CrCl(mL/min/1.73m2)	Recommended interval dosing of vancomycin
Mild 130–150	15-20 mg/kg q8-12 hr (up-to 4 gm/day)
Moderate 150–200	3–4 g/day
High ARC 200–250	4–4.5 g/day
Very high ARC 250–300	4.5–5.5 g/day
extreme ARC >300	6.0 g/day

Table 4 depicts the recommended daily vancomycin infusion dose adjustment based on eGFR [13].

**Table 4:** Recommended vancomycin infusion dose based on creatinine clearance Pea F, Furlanut M, Negri C, Pavan F, Crapis M, Cristini F, et al.: Prospectively validated dosing nomograms for maximizing the pharmacodynamics of vancomycin administered by continuous infusion in critically ill patients. Antimicrob Agents Chemother. 2009, 53:1863-7. 10.1128/AAC.01149-08 [13] eGFR: estimated glomerular filtration rate

Creatinine clearance (ml/min)	Vancomycin dosing gm/24hr
30	1.1
50	1.4
75	1.9
100	2.3
125	2.7
150	3.2
175	3.6
200	4.0
225	4.5
250	4.9
275	5.3
300	5.8
325	6.7

Many patients need dose adjustment due to the phenomenon of ARC, requiring extensive therapeutic drug monitoring (TDM) to ensure optimal therapy. In our case, vancomycin was started at 27.7 mg/kg and increased up-to ~59 mg/kg on Days 5 and 6. Despite these vancomycin doses, vancomycin levels were subtherapeutic, which led us to believe that the phenomenon of ARC is the reason for the therapeutic failure. Of note, vancomycin infusion therapy and therapeutic drug monitoring were successful strategies to achieve therapeutic vancomycin levels as observed by Udy et al., Lonsdale et al., and Goboova et al. in the setting of ARC [14-16].

Table 5 lists some case reports that report observations of ARC with vancomycin use.

**Table 5:** Observation of augmented renal clearance with vancomycin use in some case reports ARC: augmented renal clearance; eGFR: estimated glomerular filtration rate

Author/year of publication	Age of patient (years)	Clinical settings	Maximum eGFR observed (ml/min/1.73 m^2^)	Maximum vancomycin dose used	Strategies used to overcome ARC	Outcome after intervention
Udy et al. [14] (2010)	41	Intra-abdominal sepsis	177	4.5 g over 24 h (49.45 mg/kg/day)	Vancomycin infusion	Achieved therapeutic level and showed clinical improvement
Lonsdale et al. [15] (2013)	44	Subarachnoid hemorrhage with ventriculitis	375	6 g over 24 h (63 mg/kg/day)	Vancomycin infusion	Achieved therapeutic level and showed clinical improvement
Goboova et al. [16] (2015)	16	Sepsis, with severe polytrauma	339.81	6 g over 24 h (67 mg/kg/day)	Therapeutic drug monitoring and dose adjustment	Achieved therapeutic level and showed clinical improvement

The previous recommendation endorsed 15-20 mg/kg of vancomycin for severe infection, including sepsis, while recent guidelines as per the American Association of Pharmacy and the Infectious Disease Society of America (IDSA) in 2020 recommend using 40 mg/kg initial loading dose and going up to 60 mg/kg of vancomycin for severe infection [17]. Chen et al. and Irriguible TM have recommended the alternate strategies of administering prolonged or continuous infusion or switching to an alternative antibiotic, which is not renally eliminated in the case of ARC [18-19]. Conventional dose adjustment of vancomycin based on eGFR may underestimate the clearance of vancomycin. Continuous infusion of vancomycin and measurement of the urine vancomycin clearance might provide better insight. However, measurement of urine vancomycin may not be available in all settings.

## Conclusions

ARC is commonly associated with subtherapeutic vancomycin levels in critically ill patients. Early recognition of ARC is vital to reduce mortality. Despite the maximum recommended dose for severe infection like sepsis, ARC could lead to the therapeutic failure of vancomycin. Either measurement of vancomycin clearance or continuous infusion in liaison with the Infectious Disease or Pharmacy department could be a possible strategy for achieving the therapeutic level.
